# Feasibility study of a SiPM-fiber detector for non-invasive measurement of arterial input function for preclinical and clinical positron emission tomography

**DOI:** 10.1186/s40658-024-00618-2

**Published:** 2024-01-31

**Authors:** Sara de Scals, Luis Mario Fraile, José Manuel Udías, Laura Martínez Cortés, Marta Oteo, Miguel Ángel Morcillo, José Luis Carreras-Delgado, María Nieves Cabrera-Martín, Samuel España

**Affiliations:** 1 Grupo de Física Nuclear, EMFTEL and IPARCOS, Universidad Complutense de Madrid, Madrid, Spain; 2 Instituto de Investigación del Hospital Clínico San Carlos (IdISSC), Madrid, Spain; 3 Unidad de Aplicaciones Médicas de las Radiaciones Ionizantes, Centro de Investigaciones Energéticas, Medioambientales y Tecnológicas (CIEMAT), Madrid, Spain; 4 Centro Nacional de Investigaciones Cardiovasculares (CNIC), Madrid, Spain

**Keywords:** Positron emission tomography, Pharmacokinetic studies, Arterial input function, Scintillation fibers

## Abstract

Pharmacokinetic positron emission tomography (PET) studies rely on the measurement of the arterial input function (AIF), which represents the time-activity curve of the radiotracer concentration in the blood plasma. Traditionally, obtaining the AIF requires invasive procedures, such as arterial catheterization, which can be challenging, time-consuming, and associated with potential risks. Therefore, the development of non-invasive techniques for AIF measurement is highly desirable. This study presents a detector for the non-invasive measurement of the AIF in PET studies. The detector is based on the combination of scintillation fibers and silicon photomultipliers (SiPMs) which leads to a very compact and rugged device. The feasibility of the detector was assessed through Monte Carlo simulations conducted on mouse tail and human wrist anatomies studying relevant parameters such as energy spectrum, detector efficiency and minimum detectable activity (MDA). The simulations involved the use of ^18^F and ^68^Ga isotopes, which exhibit significantly different positron ranges. In addition, several prototypes were built in order to study the different components of the detector including the scintillation fiber, the coating of the fiber, the SiPMs, and the operating configuration. Finally, the simulations were compared with experimental measurements conducted using a tube filled with both ^18^F and ^68^Ga to validate the obtained results. The MDA achieved for both anatomies (approximately 1000 kBq/mL for mice and 1 kBq/mL for humans) falls below the peak radiotracer concentrations typically found in PET studies, affirming the feasibility of conducting non-invasive AIF measurements with the fiber detector. The sensitivity for measurements with a tube filled with ^18^F (^68^Ga) was 1.2 (2.07) cps/(kBq/mL), while for simulations, it was 2.81 (6.23) cps/(kBq/mL). Further studies are needed to validate these results in pharmacokinetic PET studies.

**Supplementary Information:**

The online version contains supplementary material available at 10.1186/s40658-024-00618-2.

## Introduction

Currently, most PET scans in both clinical and preclinical applications begin sometime after radiotracer injection. In this way, a static image of the distribution of the radiotracer in the body is acquired and subsequently analyzed using semi-quantitative indices. This type of analysis has some limitations including factors such as blood glucose level and radiopharmaceutical incorporation time [1]. Another way to perform PET studies is through the application of dynamic image acquisition protocols and pharmacokinetic modeling [2], allowing much more information of biological and medical interest to be extracted. However, due to their methodological complexity, these protocols have been mainly restricted to new drug development and clinical and preclinical research applications [3]. In recent years, several clinical studies [4, 5] have emerged highlighting the potential diagnostic role of kinetic modeling as it is able to provide additional information more closely related to the underlying pathology. In addition, kinetic analysis can help in monitoring response to therapy [6, 7].

Pharmacokinetic PET analysis relies on the accurate estimation of the arterial input function (AIF), which describes the time course of the radiotracer concentration in the arterial blood plasma. The AIF is essential for calculating physiological parameters, such as blood flow, metabolic rate, and receptor binding potential. Traditionally, obtaining the AIF has proven to be a challenging task, involving invasive techniques such as arterial cannulation or blood sampling, which are not only burdensome for patients but also present logistical and safety concerns.

One of the main problems associated with the clinical adoption of the kinetic model is the need to have an accurate estimate of the radiotracer concentration in arterial blood during the course of the dynamic study [3, 8]. The gold standard for measuring arterial activity is through blood sampling, either manually in discrete mode or automatically in continuous mode [9, 10]. However, this technique is invasive, time-consuming, technically complex and requires specialized personnel, so it cannot be routinely performed in clinical studies. Furthermore, the volume of blood available for sampling in the preclinical setting is very limited and requires cannulation of the femoral or carotid artery, which is terminal as the animal cannot be recovered at the end of the experiment. This is a major limitation in preclinical studies since one of the main advantages of the PET technique is that it allows for longitudinal monitoring of the animals. There are alternatives such as obtaining arterial activity through dynamic PET images, known as image-derived input function [11, 12] although the low spatial resolution of dynamic PET images limits quantification due to high noise and partial volume effect. Other method uses reference tissues [13] but a suitable region within the examined area is not always available [3]. Finally, the use of population-based AIF has been proposed which estimate the AIF from a library [14].

In recent years, several proposals have been put forward with the aim of attempting to minimize the amount of sampled blood required for animal studies by using compact models [15] or microfluidics [16, 17]. However, the time during which sampling can be performed in mice is constrained to a few minutes, it still requires the use of invasive techniques, and animals need to be killed at the end of the study. Recently, some techniques for non-invasive measurement of activity in blood have been suggested, although they are at a very preliminary stage of development [18–20] and are proposed only for studies with humans.

In this study, we conducted optimization through a combination of simulation and experimental studies on a fiber detector. Simulations were performed using phantoms designed to mimic the anatomies of both mouse tails and human wrists. The detector's capabilities were explored through Monte Carlo simulations, and a comprehensive analysis of different detector components was conducted based on experimental data.

## Materials and methods

The detector developed in this study consists of a plastic scintillation fiber of a few centimeters in length and a typical diameter of 1 mm optically coupled at one end to a silicon photomultiplier (SiPM). The fiber has a high efficiency for the detection of beta particles (positrons or electrons), while the efficiency to detect gamma photons is rather low. In that way, if placed in close proximity to a superficial vessel, beta particles reaching the fiber can be detected and the AIF could be estimated non-invasively. The sensitive volume extends along the entire length of the fiber. Thus, the linear geometry of the fiber allows for the coverage of the required extent of the monitored vessel with a single detector. First, we performed a simulation study in order to assess the detector performance under different configurations for the determination of the AIF in preclinical and clinical studies. Furthermore, we performed some experiments in order to study the detector behavior using different types of components including several scintillation fibers, coating configurations of the fiber and SiPMs. The study was performed using ^18^F and ^68^Ga isotopes which have a mean (max.) positron range in water of 0.57 (2.16) mm and 2.69 (9.06) mm, respectively.

### Simulations

We conducted Monte Carlo simulations using the PeneloPET software, which simulates the decay of isotopes and the propagation of beta and gamma particles through matter, accounting for all main physics interactions and the simulation of secondary particles. Further details can be found in the literature [21–23]. Scintillation light and SiPMs were not included in the simulations as they were studied in detail experimentally. Different geometries were defined to study the detector’s behavior in preclinical and clinical scenarios as well as to validate the results in comparison with measured data. All scenarios were tested using ^18^F and ^68^Ga isotopes. The number of simulated events was adjusted on each simulation to gather sufficient statistics (2 × 10^4^ for mouse vessels, 10^5^ for human vessels, 4 × 10^7^ for mouse background and 10^8^ for human background). Water was used to represent the soft tissues, while polystyrene was used for modeling the scintillation fibers. All the elements included in the simulation were defined inside a large air cylinder to account for possible interactions of beta particles. The simulations incorporated all primary and secondary particles including positrons, electrons and photons.

#### Simulation of mouse tail

**Fig. 1:**
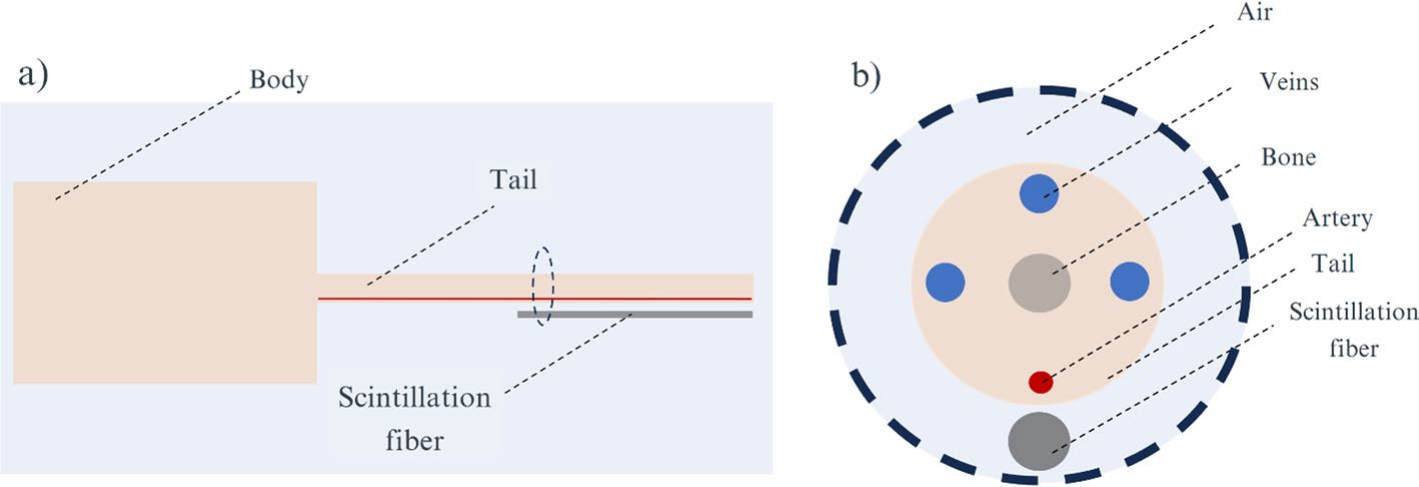
Geometry used for the simulation of the mouse anatomy. **a** Sagittal view of the simulated mouse. **b** Axial section of the tail

The capability of the detector to measure the AIF of a mouse by placing the fiber attached to its tail was studied. The tail was simulated as a water cylinder with a 3.2 mm diameter and 60 mm length including a cylindrical insert of 1 mm diameter representing the bone (see Fig. 1). The ventral tail artery was simulated as a cylinder with a diameter of 0.1 mm, and lateral and dorsal tail veins were simulated with a diameter of 0.2 mm. All vessels were located at 0.1 mm to the tail surface [24]. The mouse body was simulated as a water cylinder with a 25 mm diameter and 70 mm length and filled with 10 MBq ^18^F. The fiber with a 1 mm diameter and 30 mm length was positioned along the ventral side of the tail leaving a 0.1 mm air gap between them and at 30 mm from the body. Separate simulations were performed to record the contribution from the artery, veins and body. Energy spectra of detected events and detection efficiency are reported. The detection efficiency is defined as the percentage of detected positrons relative to the number of decays.

#### Simulation of human wrist

**Fig. 2:**
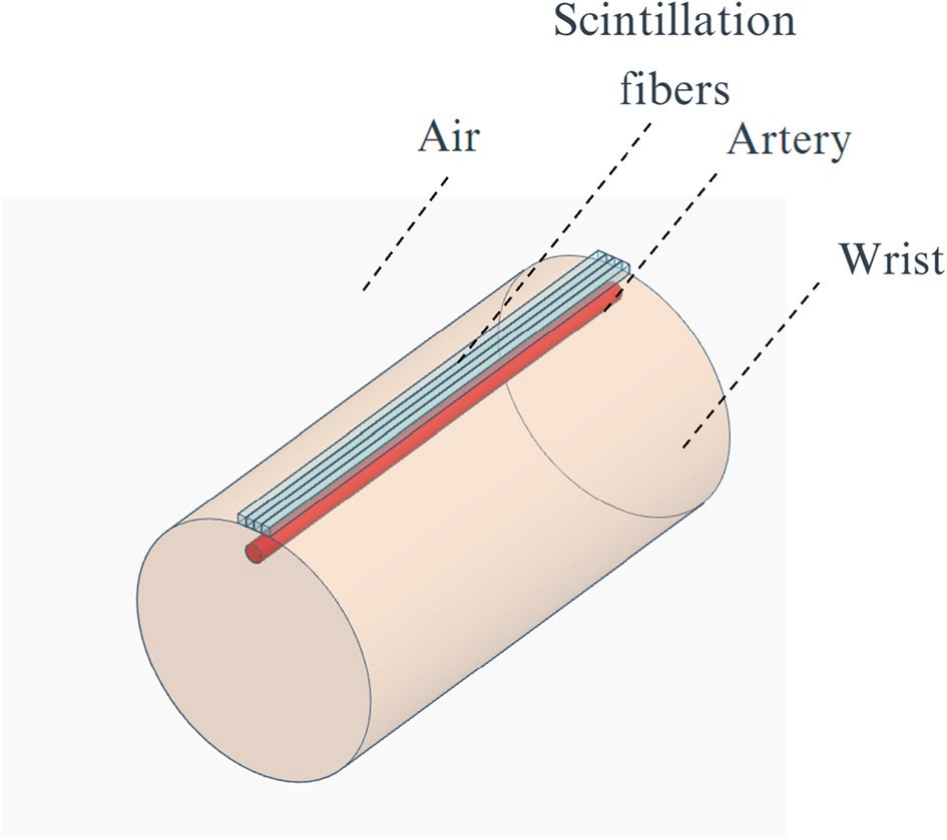
Geometry used to simulate the human wrist anatomy

The measurement of AIF in humans by placing the fiber attached to the wrist was studied. The wrist was defined as a water cylinder with 8 cm diameter and 12 cm length and the artery was modeled as a cylinder with a 2.3 mm diameter and 10 cm length (see Fig. 2). As a first approach, we tested the capability to measure AIF in humans in a reference geometry locating the artery at a depth of 2 mm and placing a single cylindrical scintillation fiber with a 1 mm diameter and 10 cm length parallel to the artery and at 0.1 mm from the skin. The artery depth was defined as the distance from the skin to the vessel surface.

Due to the larger size and deeper position of human vessels, the efficiency of detection could be increased by adding more fibers to the detectors. For that purpose, we performed simulations with several fibers with square section (from 1 to 4 fibers with 1 × 1 × 100 mm size each) placed in parallel. Furthermore, in order to study the performance of the detector as a function of the vessel depth a rectangular fiber was modeled simulating the vessel at depths ranging from 0 to 2 mm in 0.5 mm steps.

Finally, to estimate the detected background gamma radiation from the human’s body, a water cylinder was simulated with a 20 cm diameter and 70 cm length filled with 176 MBq of ^18^F and placed at 30 cm from the fiber detector.

#### Minimum detectable activity

The minimum detectable activity (MDA) is defined as the minimum amount of radioactive material necessary to determine if a source is present or not with 95% confidence [25]. MDA may be defined as

1 $${\text{MDA}}=\frac{4.65\sqrt{{N}_{{\text{B}}}+2.71}}{fTs}$$

where *f* is the branching ratio for β^+^ decays (0.9673(4) for ^18^F and 0.8914(9) for ^68^Ga), *T* is the frame duration, *N*_B_ is the amount of background events detected during *T*, and *s* is the sensitivity for the detection of positrons emitted within the artery defined as the detected count rate divided by the activity concentration in the artery. *N*_B_ and *s* were obtained from the simulations of the body and the artery, respectively, as described above for mouse and human cases. In particular, *s* was obtained by multiplying the number of detected positrons by the arterial volume subtended by the detector and dividing it by the number of simulated decays. *T* was set to 1 s for the mouse [26] and 5 s for the human [27], mimicking the frame duration at the maximum of the AIF.

### Measurements

**Fig. 3:**
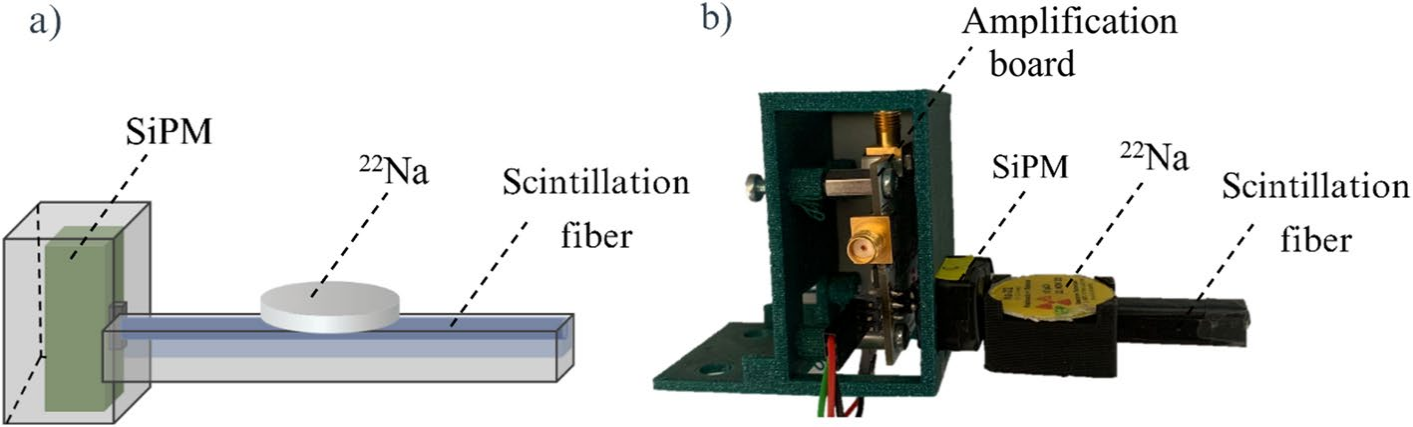
Schematic diagram (**a**) and photograph (**b**) of the detector set up consisting of a scintillation fiber coupled to a SiPM enclosed in a 3D-printed plastic housing. An encapsulated ^22^Na point source was placed on top of the detector to study the detection efficiency for each configuration

**Fig. 4:**
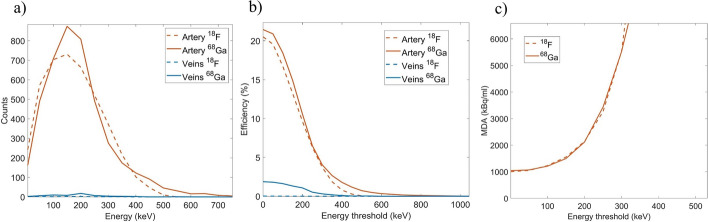
Energy spectra of events detected in the fiber (**a**), detection efficiency (**b**) and MDA (**c**) as a function of the lower energy threshold for mouse simulations involving ^18^F (dashed line) and ^68^Ga (solid line) in the ventral tail artery (orange) and the lateral and dorsal veins (blue)

Detectors with different components were assembled in order to study the optimal configuration. The device consisted of a plastic scintillation fiber with a 7 cm length coupled at one end to a SiPM (see Fig. 3) using optical grease. The SiPM was connected to an amplification board based on an inverting transimpedance amplifier (ASD-EP-EB-N, AdvanSiD, Trento, Italy) (see Fig. 4). The SiPM was operated at 31.6 V except otherwise specified. The signal from the SiPM was digitized with an oscilloscope (Picoscope Series 2206A, Pico Technology Ltd, Cambridgeshire, UK) and sent to a PC for further processing. The fiber and the SiPM were enclosed in a 3D printed plastic housing with an opening toward the detection area which was covered with thin Tedlar foil (DuPont) to minimize the attenuation of positrons and to prevent the light from entering the detector. The fiber might be coated with a reflector material to increase light collection as described below.

In order to study the efficiency for each detector configuration, a 37 kBq ^22^Na point source encapsulated in a laminated disk was used to minimize the attenuation of the positrons. The source was placed at ~ 1 mm distance from the center of the fiber (see Fig. 3). Efficiency at other longitudinal positions may yield slightly different results (less than 5%), but the central position was selected to ensure a comparison of different configurations under consistent conditions.

A graphical user interface was developed to operate the detector that allows entering the acquisition and processing parameters including the bias voltage supplied to the SiPM, the trigger level for recorded pulses and the acquisition time among others (see Additional file 1: figure S1). The output of the measurement is the count rate as a function of time and the energy spectrum of recorded events.

#### Scintillation fibers

Four scintillation fibers from Kuraray Corp. (Japan) that have different emission wavelength, shape, and number of cladding layers were tested (see Additional file 1: Table S1). All fibers have a transverse size of 1 mm with round or square shape. The fibers were left uncoated and coupled to a ASD-NUV4S-P SiPM. The energy spectra and the average count rate for 30-s acquisitions were obtained.

#### Fiber coatings

According to the technical specifications of the scintillation fibers, only a few percent of the scintillation light produced is transmitted through the fiber. In order to increase transmitted light, different coatings of the fiber were tested including black acrylic paint, white acrylic paint, teflon tape, aluminum foil and no coating (see Additional file 1: Figure S3). For this purpose, a SCSF-81J RD fiber was coupled to a ASD-NUV4S-P SiPM. The ^22^Na point source was used to perform acquisitions of 30 s and the energy spectra and the average count rate were obtained.

Differences among coatings might be due to the attenuation of the positrons when passing through them. To study this, two types of measurements were performed. First, the ^22^Na point source and a Geiger detector were placed at 1 cm distance and a thin plastic foil was located in between as a support containing different layers (from 0 to 3) of Teflon or white acrylic paint. For each case, the detected gamma photons were subtracted using additional measurements shielding the positrons with a 1 mm thick aluminum foil. Second, gamma photons from a positron-shielded source were detected with the fiber detector using different coatings.

#### Silicon photomultipliers

Four SiPMs from AdvanSiD (Trento, Italy) were tested as light sensors including devices with different active areas and peak sensitivity wavelengths (see Additional file 1: Table S2). The SiPMs were coupled to a SCSF-81J RD fiber. A study was performed to optimize the bias voltage supplied (from 28.5 to 32 V in 0.1 V steps) to the SiPM and the trigger level (from 20 to 100 mV) in 1 mV steps. For each SiPM, all measurements were repeated with and without the ^22^Na source in order to estimate the dark current for each configuration.

### Measurements validation

In order to compare the measurements obtained with the detector with Monte Carlo simulations, a specific source geometry was defined so that it would allow us to perform simulations and measurements under similar conditions. The active source was defined as a water tube with a square section of dimensions 1 × 1 × 70 mm and separated from the fiber by a 200 µm plastic wall plus 200 µm air gap. For the measurements, the tube was 3D-printed in PLA and filled with a syringe through one end. Uncoated scintillation fibers (SCSF-78J SQ for ^18^F or SCSF-78J RD for ^68^Ga) coupled to a ASD-RGB4S-P SiPM were used. The source compartment was filled with a concentration of 24,400 ± 200 and 412,410 ± 180 kBq/mL for ^18^F and ^68^Ga, respectively, and acquisitions lasted for 15 h. The detector sensitivity obtained from simulations and measurements were compared using measurements at a count rate of 1000 counts per second (cps) to avoid dead time effects. Dead time was estimated by fitting the decay curve for ^68^Ga to a non-paralyzable model. A measurement method applicable to short-lived radioisotope sources known as decaying source method was used, based on Eq. 2,

2 $$m{e}^{\lambda t}= -{n}_{0}\tau m+{n}_{0}$$

where *m* is the measured rate as a function of time, $\lambda$ is the decay constant of ^68^Ga, *t* is the elapsed time, *n*_0_ is the true rate at the beginning of the measurement, and *τ* is the dead time.

## Results

### Simulations

#### Simulation of mouse tail

Figure 4 shows the energy spectra and detection efficiency of the mouse tail simulation including independent simulations for the artery and the veins. The efficiency is depicted as a function of the applied lower energy threshold, where only events with a deposited energy above that threshold are included. All vessels were filled with the same activity concentration and the same acquisition time was simulated. Combining all vessels and in case of no energy cut, the percentage of detected positrons which were emitted within the veins was 1.24% and 5.38% for ^18^F and ^68^Ga, respectively. We must consider that there are 3 veins and only 1 artery and the diameter of the artery was half that of the vein. Therefore, there was 12 times more activity in the veins than in the artery. The percentage of detections corresponding to gamma photons was 1.62% and 0.18% for ^18^F and ^68^Ga. We can notice that even though the energy of the positrons from ^18^F and ^68^Ga are very different (*E*_max_(^18^F) = 633.5(6) keV, *E*_max_(^68^Ga) = 1899.1(12) keV), the energy spectra are very similar. This is because the thin fiber acts as a transmission detector, i.e., energetic positrons escape the fiber with remaining energy. The MDA shown in Fig. 4 was obtained using the sensitivity obtained for the artery simulation and the background events obtained for the mouse cylinder simulation. For a lower energy threshold below 100 keV a MDA close to 1000 kBq/mL was obtained which can be suitable to obtain the AIF according to published values [28]. However, we could still use longer fibers to cover a larger fraction of the artery and in many cases the artery diameter might be bigger that the one simulated in this study. Future work will include further optimization of the fiber detector in a preclinical setting.

#### Simulation of human wrist

**Fig. 5:**
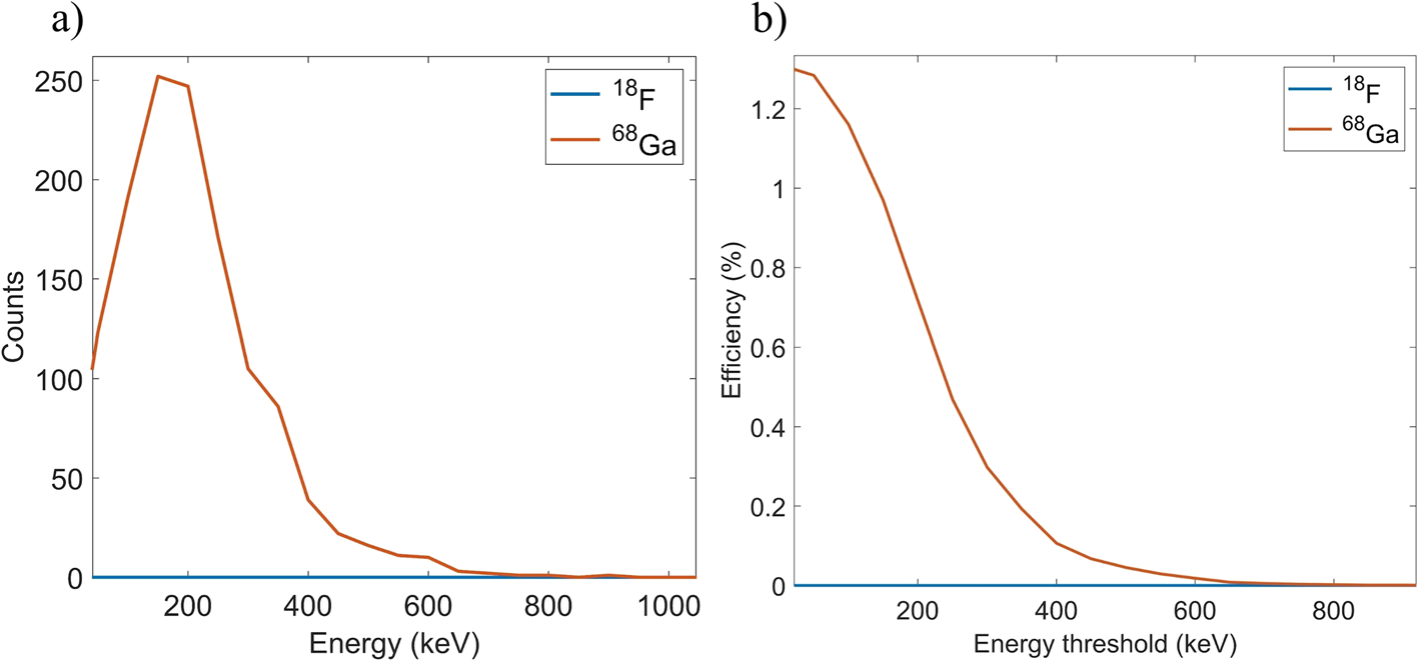
Simulated energy spectra of detected events (**a**) and detection efficiency as a function of the lower energy threshold (**b**) for simulations of the human wrist and a single cylindrical fiber detector including results for ^18^F (blue) and ^68^Ga (orange) placed in an artery at 2 mm depth. Only a few positrons from ^18^F were detected resulting in negligible counts and efficiency as depicted with blue lines

Figure 5 shows the energy spectra and the detection efficiency for the simulation on the human wrist geometry with a detector based on a single cylindrical fiber and an artery seated at 2 mm depth. The efficiency for ^68^Ga is about 20 times lower than in mouse geometry due to higher attenuation of the positrons and lower geometrical efficiency. For ^18^F, only very few positrons were detected as the maximum positron range in water [22] is 2.16 mm.

**Fig. 6:**
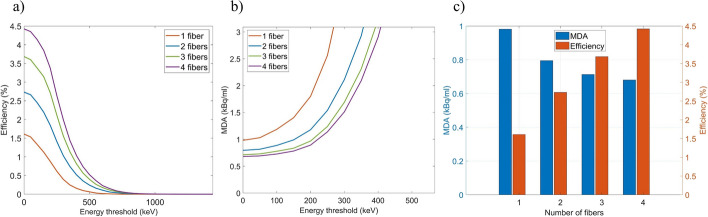
Efficiency of detected events (**a**) and MDA (**b**) as a function of the lower energy threshold for the simulation of the human wrist with an artery placed at 2 mm depth and filled with ^68^Ga. Fiber detectors containing 1 to 4 squared fibers were tested. The detection efficiency and MDA is shown with no energy cut (**c**)

The simulation for ^68^Ga in an artery placed at 2 mm depth was repeated for fiber detectors made with a different number of square fibers (1–4) as shown in Fig. 6. We observed that, while the efficiency is growing with the number of fibers, the MDA is close to saturation, as the background events increase linearly with the number of fibers while it is not the case for the detection of positrons emitted from the artery.

**Fig. 7:**
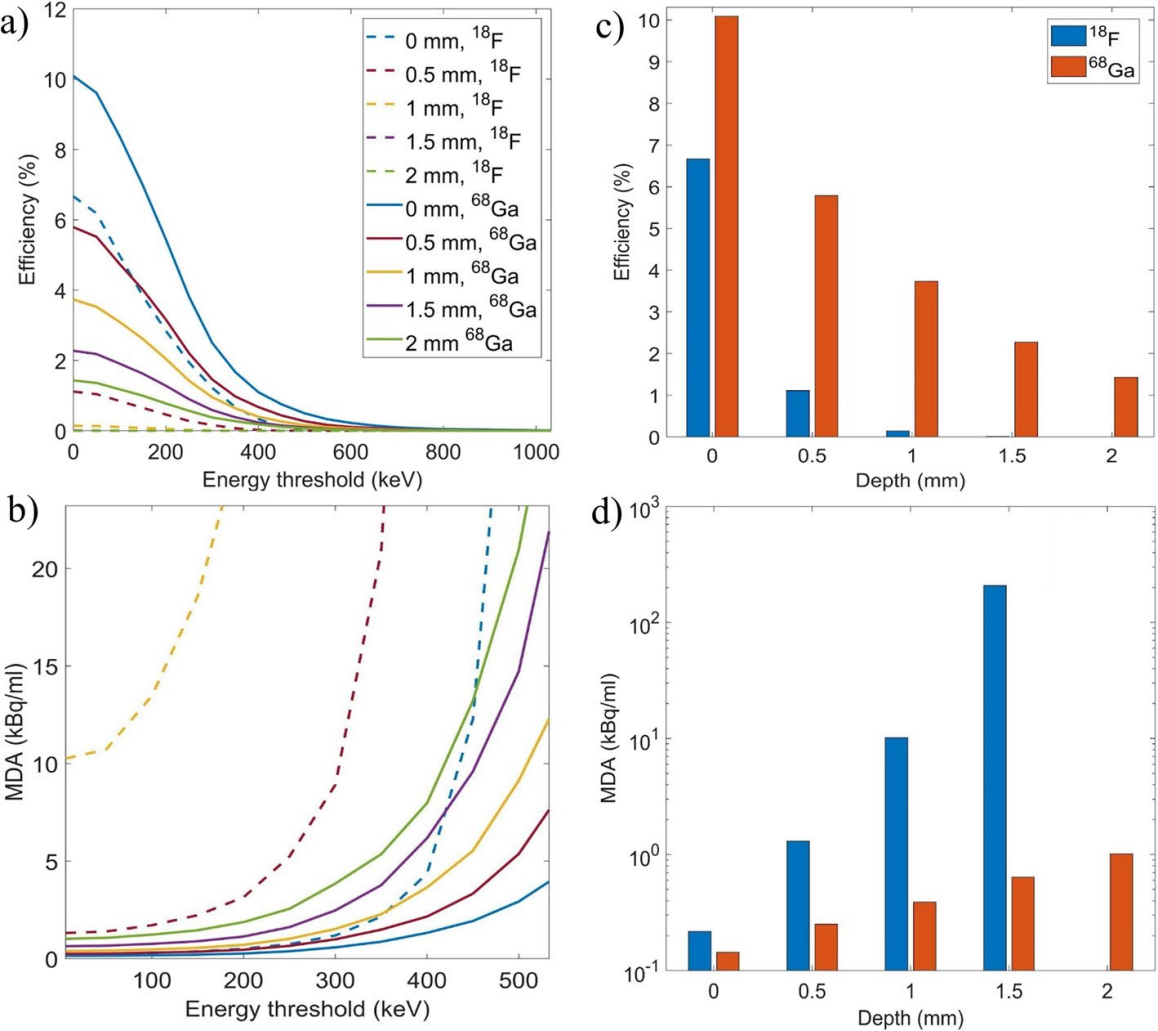
Efficiency of detected events (**a**, **c**) and MDA (**b**, **d**) for simulations of a single fiber detector in a human wrist geometry when the vessel containing ^18^F (dashed lines) or ^68^Ga (solid lines) is placed at depths ranging from 0 to 2 mm in 0.5 mm steps. Results are shown as a function of the lower energy threshold (**a**, **b**) and as a function of the vessel depth with no energy cut applied (**c**, **d**)

Additional simulations were performed to determine the effect of the vessel (could be either artery or vein) depth within the wrist on the detection efficiency and the MDA. For that purpose, a single fiber detector was simulated for vessels filled with ^18^F and ^68^Ga. Results are shown in Fig. 7. A large drop in efficiency was obtained for ^18^F which is already very low at 1 mm depth. Looking at MDA results, AIF measurements with ^18^F would be feasible for vessels up to 1 mm depth [29] although could be improved with a larger number of fibers as previously shown. In the case of ^68^Ga, suitable MDA values were obtained for depths up to 2 mm and even deeper vessels might be still suitable for AIF measurements. The energy spectra obtained for simulations with different number of fibers and different vessel depths are shown on Additional file 1: Figure S4.

### Measurements

#### Scintillation fibers

Table 1 and Additional file 1: Figure S5 show the mean count rate and energy spectra, respectively, detected using different scintillation fibers. The highest count rate was obtained for the square fiber (SQ) which has a larger sensitive volume compared to round ones (RD). Surprisingly, the fiber with multiple cladding (SCSF-78MJ RD) shows the lowest count rate (< 10%) even though according to its technical specifications it has higher light yield due to its better trapping efficiency. However, its higher numerical aperture might reduce detection efficiency at the SiPM surface for light entering at higher incident angles. Some count rate reduction might be due to positron absorption in the extra cladding layer. The energy spectrum obtained for the SCSF-81J RD fiber shows the lowest scintillation light detection, which might be due to its lower light yield compared to SCSF-78 ones according to the manufacturer.

**Table 1:** Average count rate recorded for each of the scintillation fibers tested in this study

Fiber	SCSF-81J RD	SCSF-78J RD	SCSF-78MJ RD	SCSF-78J SQ
Count rate (cps)	11,000 ± 100	11,020 ± 180	10,160 ± 170	12,080 ± 110

#### Fiber coatings

**Fig. 8:**
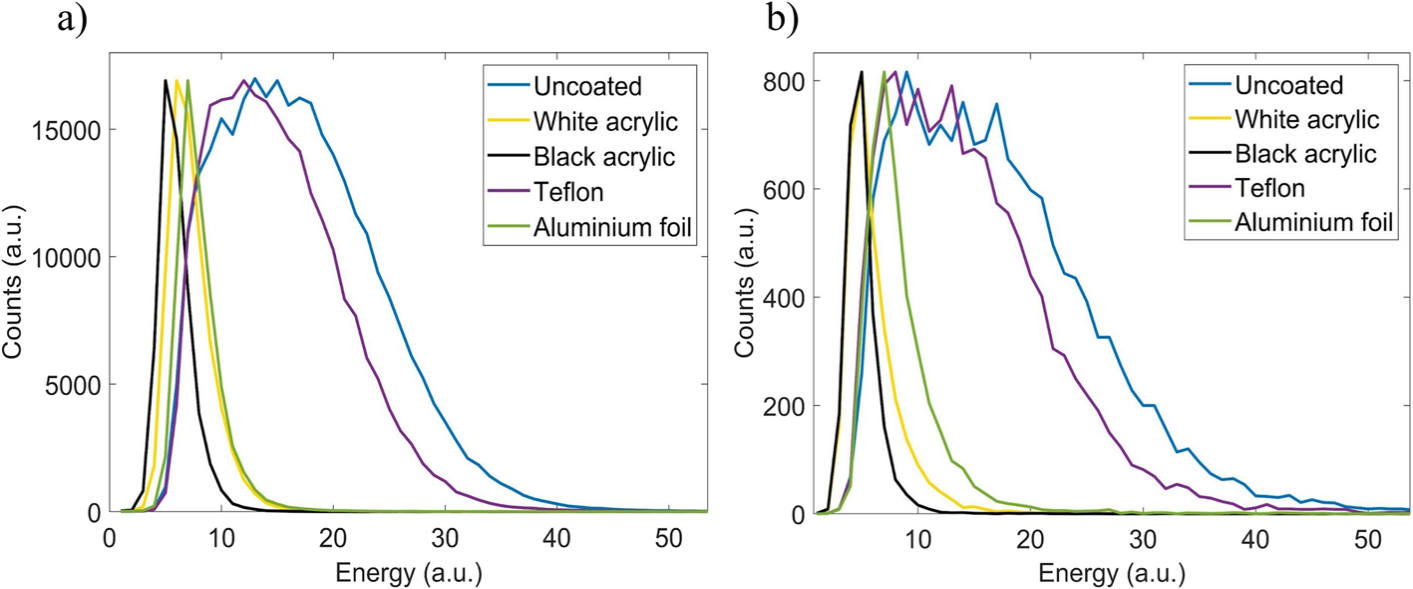
Energy spectra for measurements performed with the fiber detector using an unshielded source (**a**) or using a 1 mm thick aluminum foil to stop the positrons (**b**). Spectra are normalized to the same maximum

Table 2 and Fig. 8 show the average count rate and the energy spectra respectively recorded with the fiber detector using the SCSF-81J RD fiber covered with different coatings.

**Table 2:** Average count rate recorded with the fiber detector for each of the fiber coating materials tested in this study

Coating	Uncoated	White acrylic	Black acrylic	Teflon	Aluminum foil
Count rate *γ* + *e*^+^ (cps)	10,460 ± 90 (100%)	5300 ± 100 (51.2 ± 0.9%)	2620 ± 80 (25.1 ± 0.8%)	8710 ± 130 (83.3 ± 1.3%)	1370 ± 40 (13.1 ± 0.4%)
Count rate *γ* (cps)	523 ± 21 (100%)	440 ± 30 (84 ± 5%)	292 ± 19 (56 ± 4%)	480 ± 23 (92 ± 4%)	229 ± 17 (44 ± 3%)

The percentage of events detected with respect to uncoated fiber is shown in parenthesis. The errors were obtained as the standard deviation of three independent measurements

The attenuation of the positrons emitted from the ^22^Na source through different coatings is shown in Table 3. The number of positrons detected with the Geiger detector decreases when increasing the number of coating layers although more than 75% of the positrons can still reach the detector for 3 layers. This can explain the decrease in count rate for Teflon coating compared to uncoated fiber shown in Table 2 although the decrease in white acrylic coated fiber is significantly higher. This effect might be due to a chemical reaction between the acrylic paint and the cladding material. An alternative explanation could be attributed to the higher refractive index of the cured binder included in acrylic paints compared to air, resulting in reduced total reflection within the fiber. This explanation could be also valid for all other reflectors.

**Table 3:** Percentage of positrons emitted from the ^22^Na source that are detected in a Geiger detector after crossing different layers of coating materials compared measurements performed with no coating

# layers	White acrylic (%)	Teflon (%)
1	84.3 ± 1.1	91.5 ± 0.6
2	85.3 ± 1.4	87.6 ± 0.7
3	78.0 ± 1.0	77.8 ± 0.3

The errors were obtained as the standard deviation of three independent measurements

The decrease in scintillation photon transmission along the fiber was analyzed as a function of the coating. Figure 8 shows the energy spectra obtained with the fiber detector and the positron-shielded source corresponding to detected gamma photons for each of the coating materials. The case that yields the highest light collection corresponds to the uncoated fiber followed by the fiber coated with Teflon while the lowest light collection corresponds to acrylic paints.

#### Silicon photomultipliers (SiPMs)

**Fig. 9:**
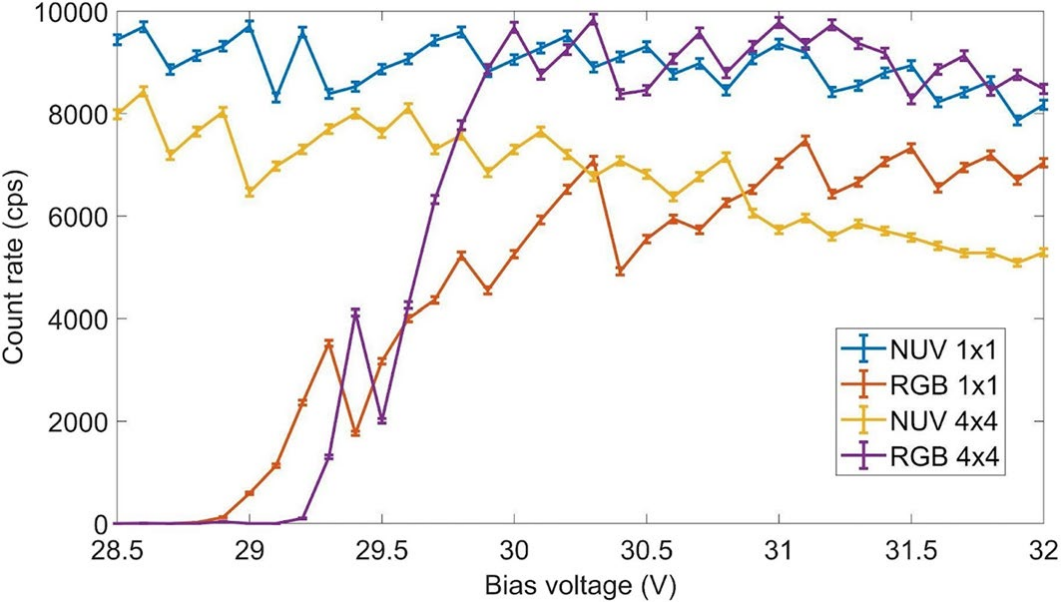
Maximum count rate as a function of the bias voltage measured for different SiPMs coupled to a scintillation fiber

Figure 9 shows the count rate obtained as a function of the bias voltage applied to each of the SiPMs under study. The count rate was obtained as the difference between count rate measured with and without the ^22^Na source. Each value corresponds to a measurement performed using the trigger level that leads to the maximum count rate. The bias voltage and trigger level that provide the maximum count rate for each SiPM are shown in Table 4. It was expected that NUV provided a better matching between SiPM PDE and the wavelength of the scintillation light emitted by the fiber. In addition, it was expected that smaller SiPMs provided better results as they have a lower number of microcells and therefore lower dark count rate while they still provide full fiber coverage. However, the best results were obtained for RBG 4 × 4 SiPM which we thought would be the worst case. Very similar results were obtained for NUV 1 × 1 SiPM.

**Table 4:** Bias voltage and trigger level that provide the highest count rate for each of the SiPMs under study

	NUV 4 × 4	RGB 4 × 4	NUV 1 × 1	RGB 1 × 1
Bias voltage (V)	28.6	30.3	29	31.1
Trigger level (mV)	24	25	25	26
Count rate (cps)	8430 ± 90	9840 ± 100	9710 ± 100	7470 ± 90

### Validation

**Fig. 10:**
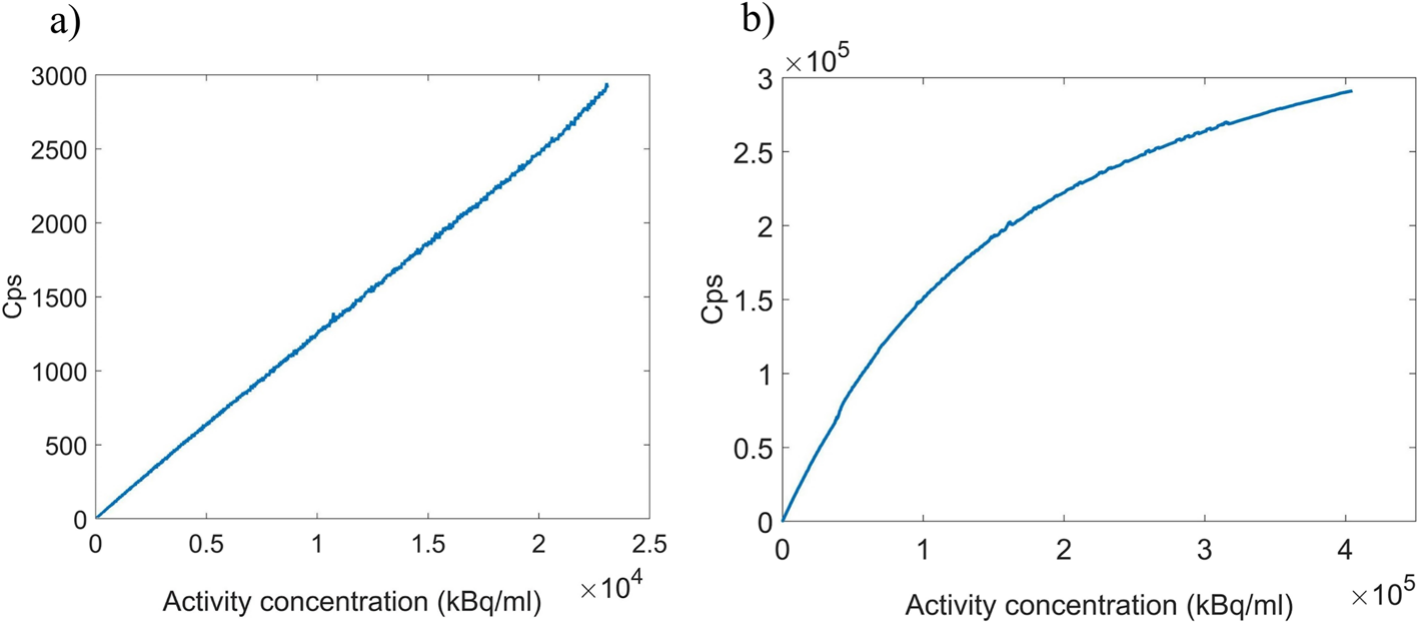
Count rate measured with a fiber detector as a function of the activity concentration within the water filled tube with ^18^F (**a**) and ^68^Ga (**b**)

Figure 10 shows the count rate obtained for 15-h measurements performed with a water filled tube containing ^18^F or ^68^Ga. The internal dimensions of the microtube are 1 × 1 × 70 mm, so it has an internal volume of 70 μL. The activity concentration was obtained by dividing the total activity by the water volume within the tube. Dead time effects were only visible for the ^68^Ga acquisition, which was used for dead time calculation by means of a non-paralyzable model. A dead time of 2.3664 ± 0.0011 μs was measured.

**Fig. 11:**
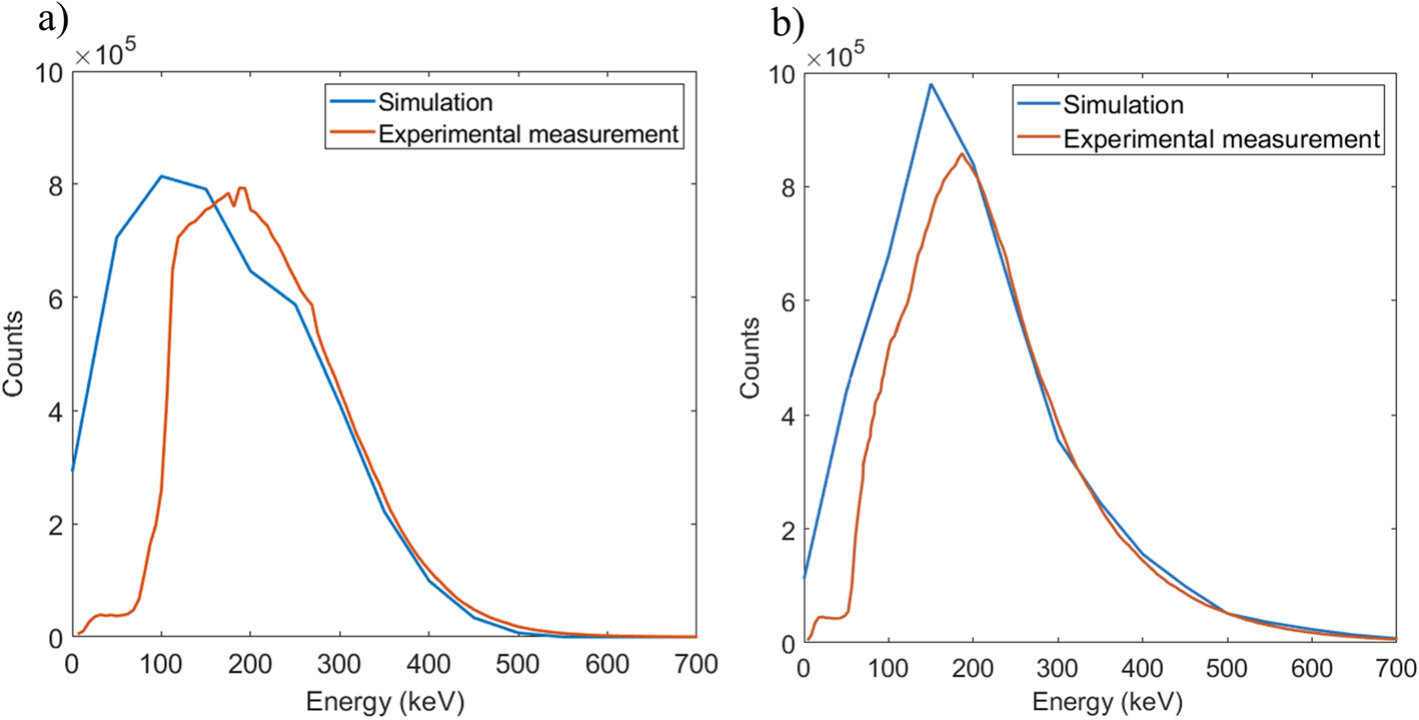
Energy spectra obtained from simulations (blue) and measurements (orange) with ^18^F (**a**) and ^68^Ga (**b**) for the validation of the fiber detector

Figure 11 shows the comparison of the energy spectra obtained from simulations and measurements performed to validate the fiber detector. The experimental spectrum was scaled horizontally and vertically to match the simulated spectrum by least squares minimization skipping the lower energies of the spectrum. A very good fit between simulated and measured spectra is shown for both ^18^F and ^68^Ga cases, suggesting that the lower energy threshold applied to the measurements is about 100 keV.

**Fig. 12:**
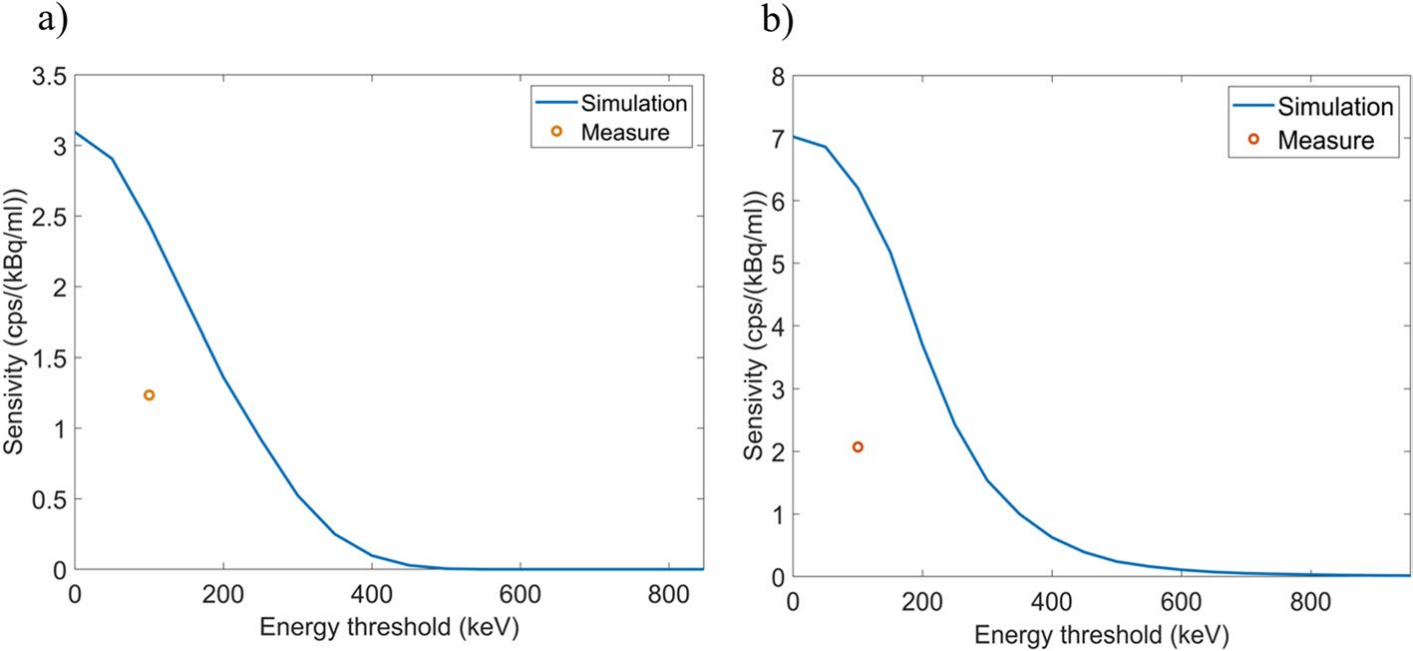
Sensitivity of the fiber detector as a function of the lower energy threshold for simulations and measurements performed with a water filled tube containing ^18^F (**a**) and ^68^Ga (**b**). The blue line refers to the simulated sensitivity as a function of the lower energy threshold and the orange dot to the experimentally measured sensitivity with an estimated lower energy threshold of about 100 keV

Figure 12 and Table 5 show the sensitivity obtained from measurements (lower energy threshold ~ 100 keV) and simulations. The simulated sensitivity is significantly higher than the measured one even considering the imposed lower energy threshold applied in measurements to avoid the dark current. Differences might be due to uncertainties in the distances between the source and the fiber and in the geometrical definition of the activity source and surrounding materials, as small deviations might have a large impact on the sensitivity.

**Table 5:** Sensitivity of the fiber detector obtained from simulations and measurements with ^18^F and ^68^Ga using a lower energy threshold of 100 keV

Source	Measurement (cps/(kBq/mL))	Simulation (cps/(kB/mL))
^18^F	1.234 ± 0.012	2.81 ± 0.05
^68^Ga	2.07 ± 0.02	6.23 ± 0.07

## Discussion

Previous studies suggested the use of detectors based on scintillation fibers. In some cases, a long scintillation fiber was rolled around the wrist and the scintillation light was read to both ends with photomultiplier tubes (PMTs) [18, 19]. This setup suffers from low efficiency as only a small portion of the fiber lies over large vessels and position discrimination relies on very accurate time difference measurements between both PMTs. Other studies [20, 30] suggested the use of a large number of straight scintillation fibers which are read individually in order to discriminate between arterial and venous blood although important parameters such as MDA were not explored. In this study, a much simpler detector is proposed that contains a single SiPM for the readout of up to 4 straight scintillation fibers. However, additional methodologies to discriminate between arterial and background positrons may still be needed, as mentioned below. Furthermore, we applied the detector not only to humans but also to preclinical studies which might also benefit from this technique.

In this study, the MDA of the detector was estimated by considering only the background generated by gamma photons emitted from the body. However, additional background events may arise from positrons emitted by other tissues, such as the vessel wall and the skin. Therefore, forthcoming studies will involve animals and humans to compare the AIF obtained with the detector against manually drawn blood samples. The background from other tissues will be thoroughly evaluated, and strategies to discriminate between positrons emitted from the arteries and those from other tissues will be explored.

The proposed detector would require a single point calibration in order to convert the recorded count rate to activity concentration. For that purpose, a blood sample or a value derived from PET images would be required. An alternative approach involves creating a detailed anatomical model of the patient, which can be obtained through imaging techniques such as ultrasounds, and simulate the propagation of the positrons using Monte Carlo tools [30]. An important challenge in positioning the fiber on real mouse tails and human wrists is accounting for possible movements. Therefore, ensuring a secure coupling of the detector to the subject is crucial to prevent variations in sensitivity during data acquisition.

## Conclusions

In this study, a detector based on scintillation fibers and SiPMs for non-invasive measurement of the AIF in preclinical and clinical PET pharmacokinetic studies was proposed. The feasibility of this technique was studied using Monte Carlo simulations for mouse tail and human wrist anatomies obtaining MDA values that suggest its possible application in both scenarios. Different components and configurations of the detector were studied on prototypes including different scintillation fibers, fiber coatings, SiPMs, supplied voltages and trigger levels. In conclusion, the results presented in this study allow us to move forward to consider the fiber detector as a promising tool for the non-invasive measurement of the AIF in mice and humans. Hence, additional refinements and validations will be undertaken in the near future to enable the use of these detectors in PET pharmacokinetic studies.

### Supplementary Information

**Additional file 1**. Additional figures a tables.

## Data Availability

The datasets used and/or analyzed during the current study are available from the corresponding author upon reasonable request.

## Abbreviations

PET: Positron emission tomography
AIF: Arterial input function
SiPM: Silicon photomultiplier
MDA: Minimum detectable activity
SQ: Square
RD: Round
PS: Polystyrene
PMMA: Polymethylmethacrylate
FP: Fluorinated polymer
PLA: Polylactic Acid
CPS: Counts per second
