# Correction: Feasibility study of a SiPM-fiber detector for non-invasive measurement of arterial input function for preclinical and clinical positron emission tomography

**DOI:** 10.1186/s40658-024-00624-4

**Published:** 2024-03-04

**Authors:** Sara de Scals, Luis Mario Fraile, José Manuel Udías, Laura Martínez Cortés, Marta Oteo, Miguel Ángel Morcillo, José Luis Carreras-Delgado, María Nieves Cabrera-Martín, Samuel España

**Affiliations:** 1 Grupo de Física Nuclear, EMFTEL and IPARCOS, Universidad Complutense de Madrid, Madrid, Spain; 2 Instituto de Investigación del Hospital Clínico San Carlos (IdISSC), Madrid, Spain; 3 Unidad de Aplicaciones Médicas de las Radiaciones Ionizantes, Centro de Investigaciones Energéticas, Medioambientales y Tecnológicas (CIEMAT), Madrid, Spain; 4 Centro Nacional de Investigaciones Cardiovasculares (CNIC), Madrid, Spain

Correction: *EJNMMI Phys***11**, 12 (2024). 10.1186/s40658-024-00618-2

Following publication of the original article [1], the authors requested to update the Funding section.

The original Funding sections was:

This work was funded by the Universidad Complutense de Madrid under Project PR27/21 − 009 and by the Ministerio de Ciencia e Innovación under Project TED2021-129933B-C21. S. España is supported by the Ministerio de Ciencia, Innovación y Universidades under Ayudas Ramón y Cajal RYC2018-024495-I. The CNIC is supported by the Instituto de Salud Carlos III (ISCIII), the MCIN and the Pro CNIC Foundation, and is a Severo Ochoa Center of Excellence (Grant CEX2020-001041-S funded by MICIN/AEI/10.13039/501100011033).

The correct Funding section should read:

This work is part of the Project TED2021-129933B-C21 funded by the MCIN/AEI/10.13039/501100011033 and the European Union NextGenerationEU/ PRTR as well as by the Universidad Complutense de Madrid under Project PR27/21-009. S. España is supported by the Ministerio de Ciencia, Innovación y Universidades under Ayudas Ramón y Cajal RYC2018-024495-I. The CNIC is supported by the Instituto de Salud Carlos III (ISCIII), the MCIN and the Pro CNIC Foundation, and is a Severo Ochoa Center of Excellence (Grant CEX2020-001041-S funded by MICIN/AEI/10.13039/501100011033).

The original article [1] has been updated.
