# Supplementary material for: Feasibility study of a SiPM-fiber detector for non-invasive measurement of arterial input function for preclinical and clinical positron emission tomography
Source: EJNMMI Phys. 2024 Jan 31;11:12. doi: 10.1186/s40658-024-00618-2 (PMC10828322; doi:10.1186/s40658-024-00618-2)
Supplement: Supplementary file 1 — Additional file 1. Additional figures a tables. [file 40658_2024_618_MOESM1_ESM.pdf]

Supplementary material

Feasibility study of a SiPM-fiber detector for non-invasive measurement of arterial input function for preclinical and clinical positron emission tomography

Sara de Scals[1,2], Luis Mario Fraile[1,2], José Manuel Udías[1,2], Laura Martínez Cortés[3], Marta Oteo[3], Miguel Ángel Morcillo[3], José Luis Carreras-Delgado[2], María Nieves Cabrera-Martín[2], Samuel España[1,2,4,]*

[1]Grupo de Física Nuclear, EMFTEL & IPARCOS, Universidad Complutense de Madrid

[2]Instituto de Investigación del Hospital Clínico San Carlos (IdISSC), Madrid, Spain

[3]Unidad de Aplicaciones Médicas de las Radiaciones Ionizantes, Centro de Investigaciones Energéticas, Medioambientales y Tecnológicas, (CIEMAT), Madrid, Spain

[4]Centro Nacional de Investigaciones Cardiovasculares (CNIC), Madrid, Spain

[*]Corresponding author. Email: sespana@ucm.es

Figures

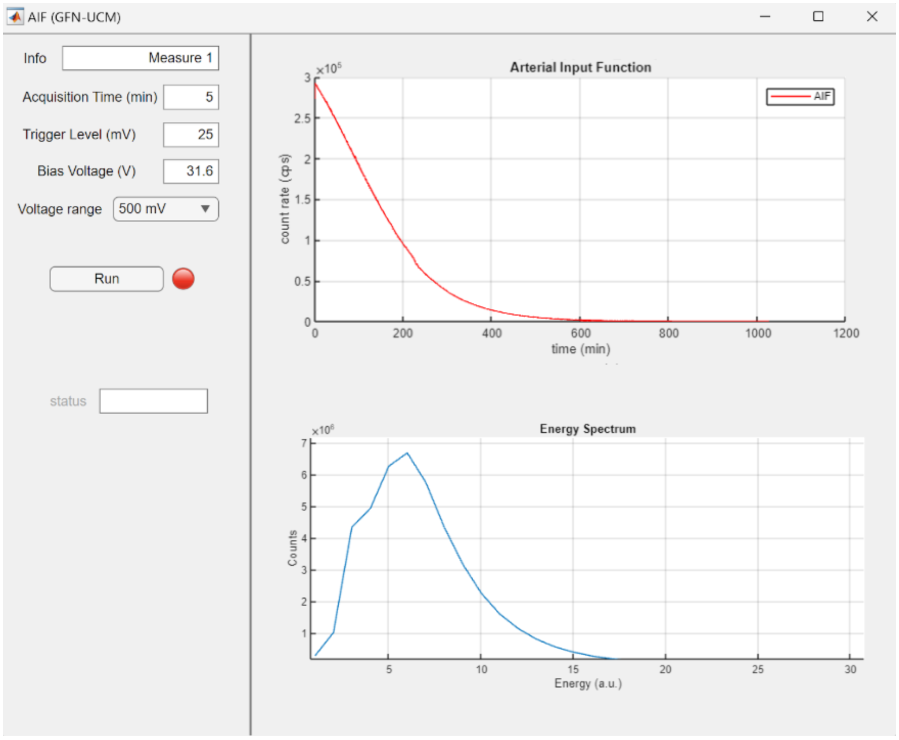

Figure S1. Graphical user interface developed to operate the detector. Graphs show the count rate and energy spectrum obtained from a 20 hours long acquisition using the microtube filled with ^{68}Ga.

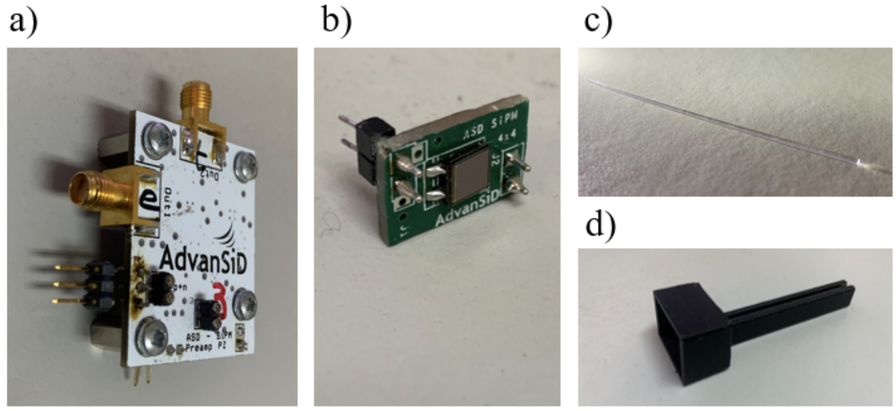

Figure S2. Main components of the experimental set up: amplification board (a), silicon photomultiplier (b), scintillation fiber (c), the plastic housing (d).

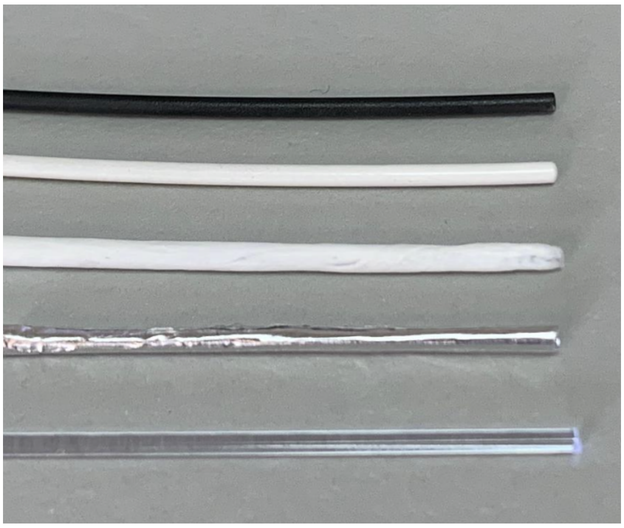

Figure S3. Different fiber coatings tested including from top to bottom black acrylic paint, white acrylic paint, teflon tape, aluminum foil and no coating.

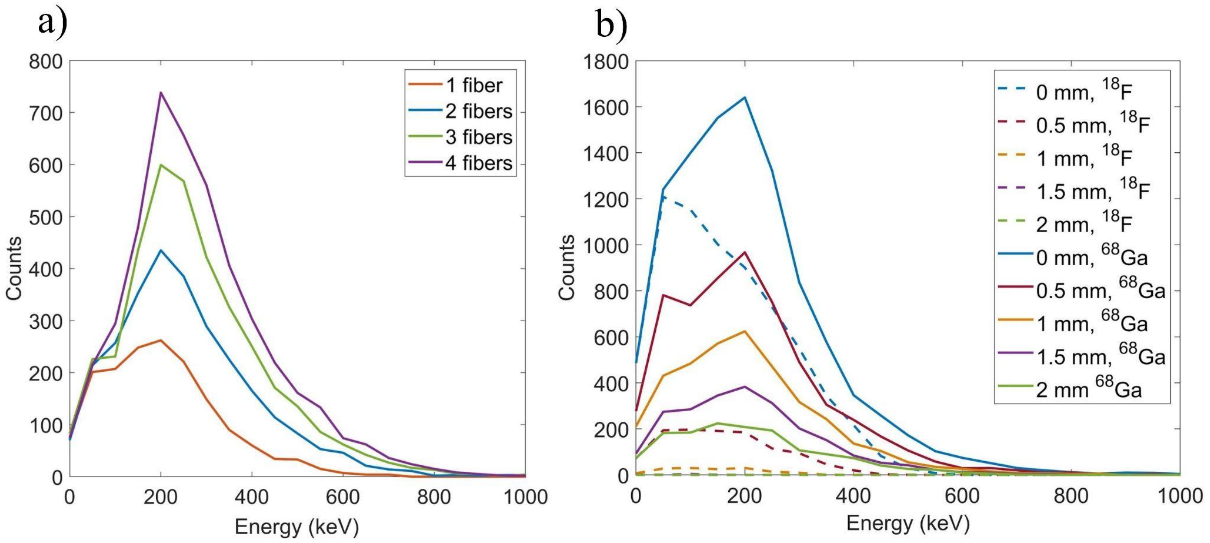

Figure S4. Energy spectra of detected events for the simulation of a human wrist geometry for a detector with different number of square fibers and the artery placed at 2 mm depth filled with ^{68}Ga (a) and for a single fiber and a vessel placed at different depths (b) and filled with ^{18}F (dashed lines) or ^{68}Ga (solid lines).

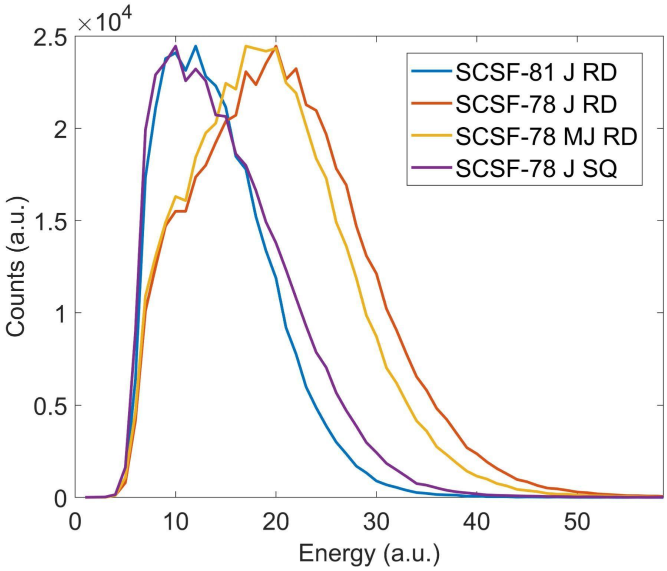

Figure S5. Energy spectra normalized to the maximum recorded for each of the scintillation fibers tested in this study.

Tables

Table S1. Main characteristics of the plastic scintillation fibers tested in this study.

Name	Emission peaks (nm)	Geometry	Core material	Cladding	Cladding material
**SCSF-81 J RD**	437	Round (RD)	Polystyrene (PS)	Simple cladding	Polymethylmethacrylate (PMMA)
**SCSF-78 J RD**	450	RD	PS	Simple cladding	PMMA
**SCSF-78 J SQ**	450	Square (SQ)	PS	Simple cladding	PMMA
**SCSF-78 MJ RD**	450	RD	PS	Multiple cladding	PMMA & Fluorinated polymer (FP)

Table S2. Main characteristics of the SiPMs tested in this study.

Name	Type	Active area (mm^2)	Cell count	Peak sensitivity wavelength (nm)
**ASD-NUV1S-P**	NUV	1×1	625	420
**ASD-NUV4S-P**	NUV	4×4	9340	420
**ASD-RGB1S-P**	RGB	1×1	625	550
**ASD-RGB4S-P**	RGB	4×4	9340	550
